# Seasonality in telomerase activity in relation to cell size, DNA replication, and nutrients in the fat body of *Apis mellifera*

**DOI:** 10.1038/s41598-020-79912-9

**Published:** 2021-01-12

**Authors:** Justina Koubová, Michala Sábová, Miloslav Brejcha, Dalibor Kodrík, Radmila Čapková Frydrychová

**Affiliations:** 1 Biology Centre of the Czech Academy of Sciences, Institute of Entomology, Branišovská 31, 370 05 České Budějovice, Czech Republic; 2 Faculty of Science, University of South Bohemia, České Budějovice, Czech Republic

**Keywords:** Ageing, Telomeres, Entomology

## Abstract

In honeybees (*Apis mellifera*), the rate of aging is modulated through social interactions and according to caste differentiation and the seasonal (winter/summer) generation of workers. Winter generation workers, which hatch at the end of summer, have remarkably extended lifespans as an adaptation to the cold season when the resources required for the growth and reproduction of colonies are limited and the bees need to maintain the colony until the next spring. In contrast, the summer bees only live for several weeks. To better understand the lifespan differences between summer and winter bees, we studied the fat bodies of honeybee workers and identified several parameters that fluctuate in a season-dependent manner. In agreement with the assumption that winter workers possess greater fat body mass, our data showed gradual increases in fat body mass, the size of the fat body cells, and Vg production as the winter season proceeded, as well as contrasting gradual decreases in these parameters in the summer season. The differences in the fat bodies between winter and summer bees are accompanied by respective increases and decreases in telomerase activity and DNA replication in the fat bodies. These data show that although the fat bodies of winter bees differ significantly from those of summer bees, these differences are not a priori set when bees hatch at the end of summer or in early autumn but instead gradually evolve over the course of the season, depending on environmental factors.

## Introduction

Eusociality is the most complex form of sociality, in which colony members are divided into reproductive and nonreproductive castes, only one or a few reproductive individuals are present in each colony, and overlapping generations of nonreproductive individuals cooperatively care for offspring^1^. Eusocial insect species offer excellent systems for aging research, as they exhibit notable lifespan differences among different castes and species and the absence of the common life history tradeoff between longevity and fecundity as reproductives, such as kings and queens, typically have longer lifespans than workers or soldiers^2–4^. Comparative analysis of the average lifespans of adult eusocial insect reproductives, such as ants, termites, and honeybees, to that of adult solitary insects has shown that the evolution of eusociality is associated with a 100-fold increase in insect longevity. The mean average lifespan of a honeybee queen is 5.6 years, while that of a solitary insect species is only 0.1 ± 0.2 years^5^.

Honeybees display remarkable plasticity in the aging rate of workers in response to the social context of the colony. In a honeybee colony, there is an age-related division of labor; young workers (referred to as nurse bees) are engaged in tasks inside the colony, while workers older than 18–28 days are responsible for foraging. In response to social-environmental stimuli, such as colony age demographics, this division of labor is accelerated, decelerated, or reversed, which is accompanied by changes in both behavioral ontogeny and aging rate and is directed by pheromone communication^6^. The transition from nursing to foraging is associated with changes biomarkers of aging, such as decreased vitellogenin (Vg) levels, increased juvenile hormone (JH) levels, and a loss of hemocytes^7–9^. Through social manipulation, workers can revert from foraging to nursing, which is a behavioral reversion that is accompanied by changes in the senescence markers^10^. The remarkable lifespan extension of winter bees is an adaptation to the cold season when the resources required for the growth and reproduction of colony are limited^10^. Winter bees do not display foraging activity, and they have excessive Vg levels in their fat bodies, elevated oxidative stress defense, and low JH levels^9,11^.

Although several theories have been proposed to explain the mechanism of aging, none of the current theories are sufficient to explain the phenomenon. However, it is widely thought that the regulation of aging and lifespan is associated with telomeres and telomere maintenance. Telomeres are specialized nucleoprotein structures located at chromosome ends that play several vital functions in cells. Telomeres distinguish natural chromosome termini from chromosome breaks and compensate for chromosome shortening^12,13^. In the absence of telomere maintenance, the chromosome ends are shortened during each successive round of cell division, ultimately leading to cell senescence or apoptosis. Normal human somatic cells have limited replication potential, and telomere shortening provides a molecular clock that determines the replicative lifespan^14,15^.

Telomere shortening is mostly compensated by the activity of telomerase, an enzyme that binds to chromosome ends and directly synthesizes new telomeric DNA on the chromosome termini^16–18^. Telomerase activity is related to cell proliferation, and the highest telomerase activity in adult individuals is detected in highly proliferating cells^19,20^. In human adults, telomerase is downregulated in most somatic cells, and in these cells, telomere length is heterogeneous and typically declines with age. Progressive telomere shortening is believed to have developed as a tumor-suppressing mechanism, but it is also linked to aging, higher mortality risk, and numerous degenerative diseases^21–23^. A decline in telomerase activity and subsequent telomere shortening may contribute to the limited division potential of stem cells, which in turn limits tissue and organ regeneration capacity, resulting in progressive aging. Telomere attrition and declining telomerase activity are believed to be hallmarks of aging and predictors of lifespan^24–27^.

Similar to vertebrates, insects display a decline in telomerase activity with advancing organismal development and age^28^. Our previous study revealed that telomerase activity is upregulated in the somatic tissues of long-lived reproductives of honeybees^29^ and termites (unpublished data), suggesting that upregulation of telomerase might be a clue to explain the extreme longevity of reproductives of eusocial species.

To extend the knowledge regarding the prolonged lifespan of winter bees of *A. mellifera*, this study was initially aimed at comparing telomerase activity between summer and winter honeybee workers. Since our data revealed that telomerase activity is strongly upregulated in the fat bodies of winter bees and is accompanied by upregulated DNA replication, we focused on obtaining additional information regarding the reason for telomerase upregulation.

## Results

### Telomerase activity is increased in winter bees

**Figure 1:**
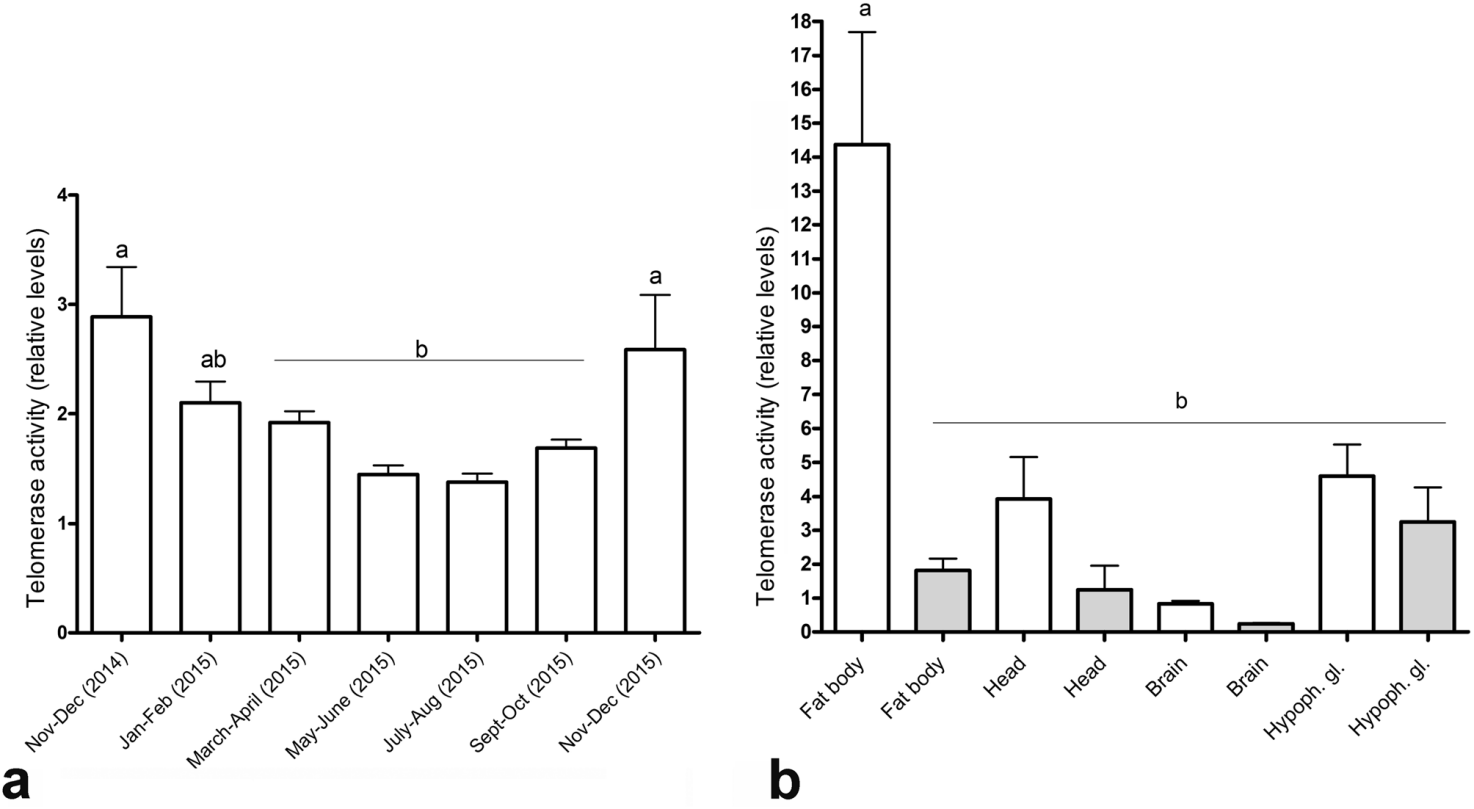
Telomerase activity levels. Relative telomerase activity was quantified using a TRAP assay in protein extracts prepared from workers collected over the course of a year **(a)**, and the dissected tissues of workers collected in February (white columns) and June (grey columns) **(b)**. Statistical significance was determined using one-way ANOVA and Tukey’s post-hoc tests (*P* < 0.05, indicated by different letters above the columns); n = 10. Bars in graphs represent the mean ± SD.

First, we performed a pilot experiment in which we measured telomerase activity in extracts prepared from workers collected in January and June, and found that telomerase activity levels in January bees were approximately twice as high as those in June bees. The specificity of the generated Telomeric Repeat Amplification Protocol (TRAP) products for (TTAGG)n repeats was confirmed by cloning and sequencing and visualization on 12% nondenaturing polyacrylamide gel (Fig. S1). In the next step, we extended our evaluation of telomerase activity to workers collected at approximately 2-month intervals over an 18-month period. The results showed a gradual increase in telomerase activity during autumn, a peak in winter samples collected from November to February, and a gradual decrease during spring. The lowest telomerase activity was detected in the summer samples (Fig. 1a). To reveal more details about the differences between winter and summer bees, we compared telomerase activity in various organs, such as the abdominal fat bodies, hemolymph, gut, brain, hypopharyngeal gland, and the whole head, in February and June bees. No telomerase activity (no Ct value) was detected in the hemolymph, the Ct values detected in the gut showed high variability between individual samples (data not shown), and no differences in telomerase activity in the hypopharyngeal gland were detected between the two groups. However, in February bees, higher telomerase activity levels were detected in extracts from whole heads (threefold; *P* < 0.5), brains (2.5-fold; *P* < 0.5), and especially fat bodies (tenfold higher levels; *P* < 0.1; Fig. 1b).

### DNA replication is increased in the fat bodies of winter bees

**Figure 2:**
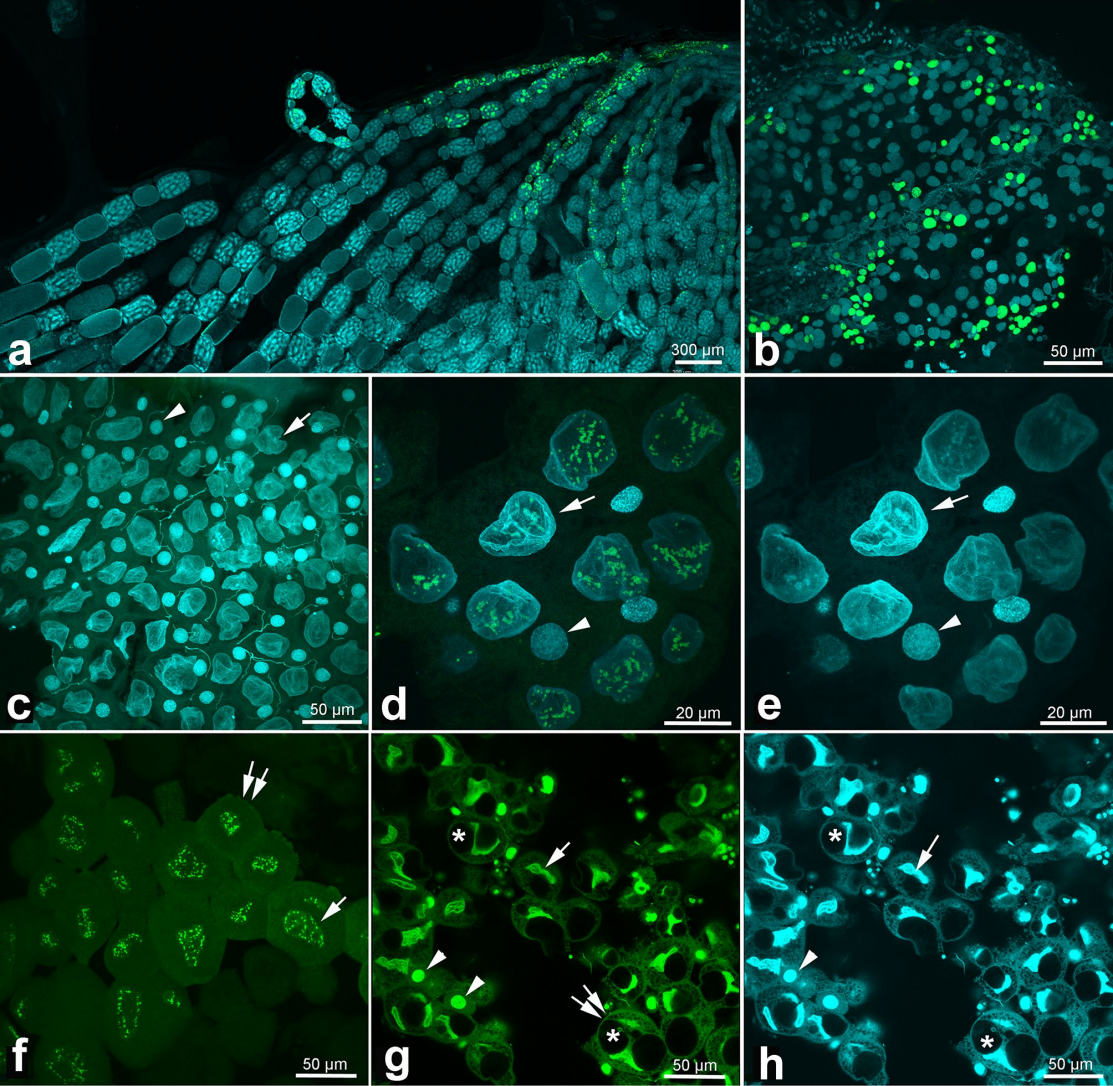
S-phase nuclei in fat bodies visualized using EdU. S-phase nuclei were visualized using the thymidine analogue EdU, which is incorporated into newly synthesized DNA and fluorescently labeled with Alexa 488 (green signals). DNA was labeled with DAPI (blue signals). The assay was performed on the ovaries of queens **(a)**, worker’s guts **(b)**, foragers in August **(c-e)**, and workers in December–January **(f–h)**. Arrows, trophocyte nuclei; arrowheads, oenocyte nuclei; double arrows, trophocytes; stars, lipid droplets.

**Figure 3:**
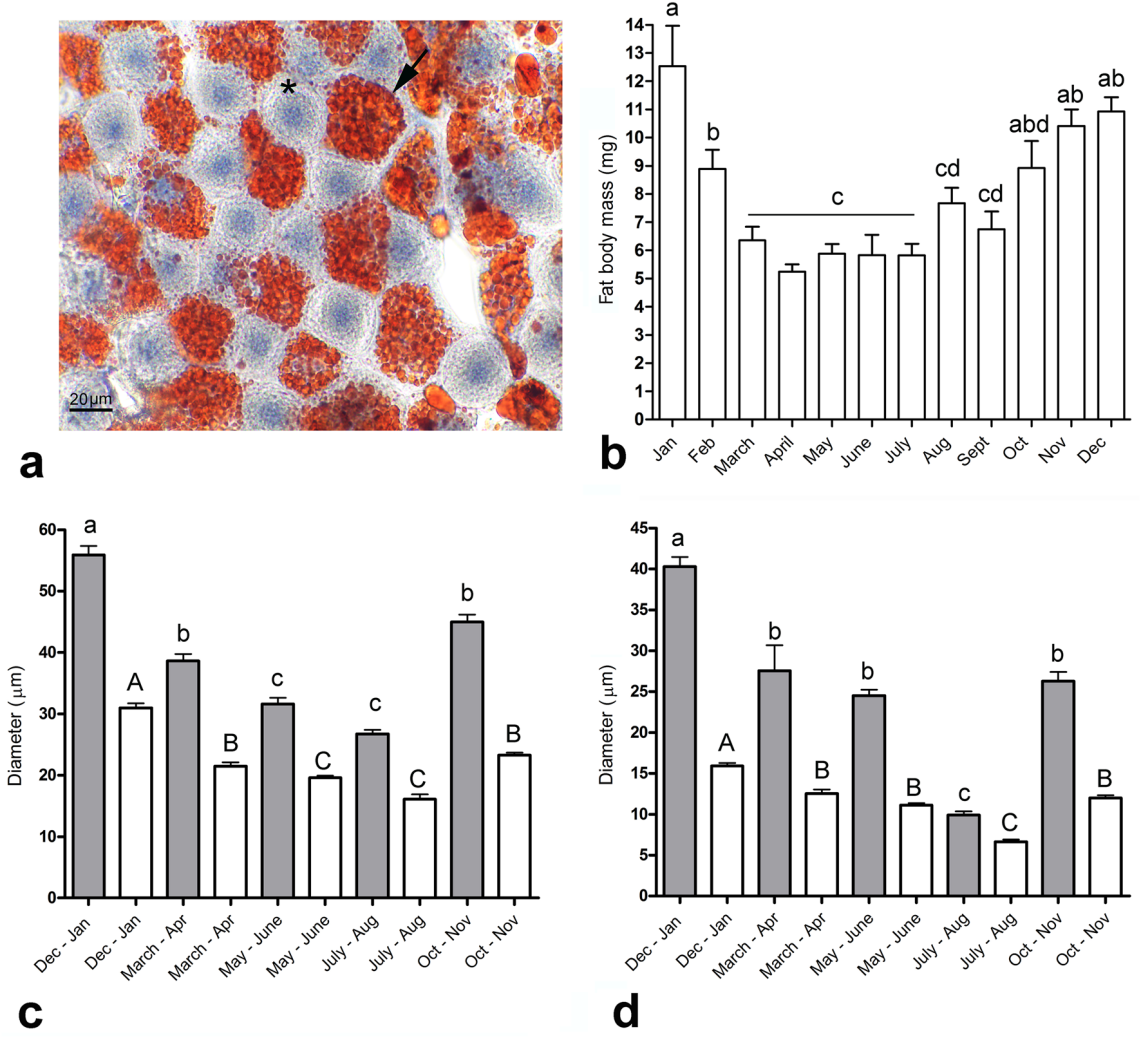
Seasonal changes in the morphology of the fat body. (**a)** The main cell types in the fat body are trophocytes and oenocytes. Trophocytes function as energy reserves, and lipids are deposited in numerous droplets in the cytoplasm (visualized with Oil Red staining, in red). Trophocytes and their nuclei tend to be irregularly shaped, whereas oenocytes and their nuclei (visualized using hematoxylin, in blue) are rounded. In workers collected throughout the year, the fat body mass of individual bees **(b)**, size of trophocytes and their nuclei **(c)**, and size of oenocytes and their nuclei **(d)** were evaluated. Cell and nuclear sizes in fat body samples counterstained with DAPI and visualized using confocal microscopy were evaluated using Adobe Photoshop. The average sizes of the cells and their nuclei are shown as grey and white columns, respectively. In March–October, experiments were performed on foragers of random age, and during the cold season (November–February), bees were sampled by random collection inside hives. Statistical significance was determined using one-way ANOVA and Tukey’s post-hoc tests (*P* < 0.05, indicated by different letters above the columns); n = 5 for the fat body mass evaluation, n > 200 for the evaluation of cell and nuclei size. Bars in graphs represent the mean ± SD.

The next question we asked was whether upregulation of telomerase activity is correlated with increased DNA replication. To visualize DNA replication, we used EdU incorporation. In a pilot experiment, we stained the ovaries of egg-laying queens and the guts of workers, which are two highly proliferative tissues, and both tissues showed strong EdU signals (Fig. 2a,b). Further experiments were conducted using workers collected at 1–2–month intervals over the course of a year, in which we assessed DNA replication in head organs (the brain and hypopharyngeal glands) and the abdominal fat bodies, using guts as a positive control. From March to October, experiments were conducted on 1-day-old bees and foragers of random age. During the cold season (November to February), bees were sampled by random collection inside hives. Consistent DNA replication was observed in the guts of all tested bees, without any significant differences in the localization or intensity of the EdU signal. Although no EdU signals were observed in the head organs of any tested sample, DNA replication was detected in the fat body, and it varied depending on the age of the bee, season, and cell type. The fat body consists mostly of two cell types, trophocytes, with irregularly shaped nuclei and abundant lipid droplets in the cytoplasm, and oenocytes, which are smaller and have roundish nuclei (Figs. 2, 3a). In April–August (Fig. 2c–e), DNA replication signals were observed only rarely in trophocytes of young bees (Fig. 2d,e). However, DNA replication signals were more frequently detected during the cold season. In October–November, the occurrence of EdU signals was found sporadically in trophocytes of around 20% of tested bees. In December and January, around 80% of the tested bees had frequent, large spots of EdU-positive cells (Fig. 2f–h). In March–November, EdU signals were detected only in trophocytes, and the intensity of EdU signals in individual nuclei was variable but overall weaker than in December-January samples. In December and January, the intensity of EdU signals in individual cells tended to be stable and was detected in both trophocytes and oenocytes (Fig. 2g).

Collectively, the results of these experiments showed that DNA replication levels in the fat body of workers oscillate throughout the year and are increased during the cold season, providing an explanation for the upregulated telomerase levels in the fat body, as indicated in previous experiments. However, we failed to observe any DNA replication in the brain or whole head; thus, the reason for the elevated levels of telomerase activity in the head samples remains to be determined.

### Are the levels of telomerase activity and DNA replication correlated with telomere length, cell and nuclei sizes and fat body mass?

Based on the polyploid character of fat body cells, we assumed that the DNA synthesis signals observed in our experiments are associated with DNA endoreduplication cycles instead of cell proliferation. Therefore, we measured the sizes of the fat body cells and their nuclei over time, and found that the sizes varied depending on the season (Fig. 3). In samples collected in December and January, the average diameters of trophocytes and trophocyte nuclei were 55 ± 13 µm and 30 ± 7.7 µm, respectively, and the average diameters of oenocytes and oenocyte nuclei were 40 ± 9 µm and 15 ± 2.6 µm, respectively. In samples collected during July and August, the average diameters were 28 ± 5.1 µm and 18 ± 3.9 µm for trophocytes and their nuclei, respectively, and 10 ± 1.7 µm and 7 ± 1.1 µm for oenocytes and their nuclei, respectively. Moreover, these sizes gradually decreased as the warm season proceeded and gradually increased as the cold season proceeded. The same trends were observed when we quantified the fat body mass in individual bees, which was 5.8 ± 1.2 mg per bee in July and 12 ± 4.0 mg per bee in January (Fig. 3b). These data collectively indicate that during the cold season, the fat body cells are up to twice as large as in the warm season, resulting in a twofold increase in fat body mass.

**Figure 4:**
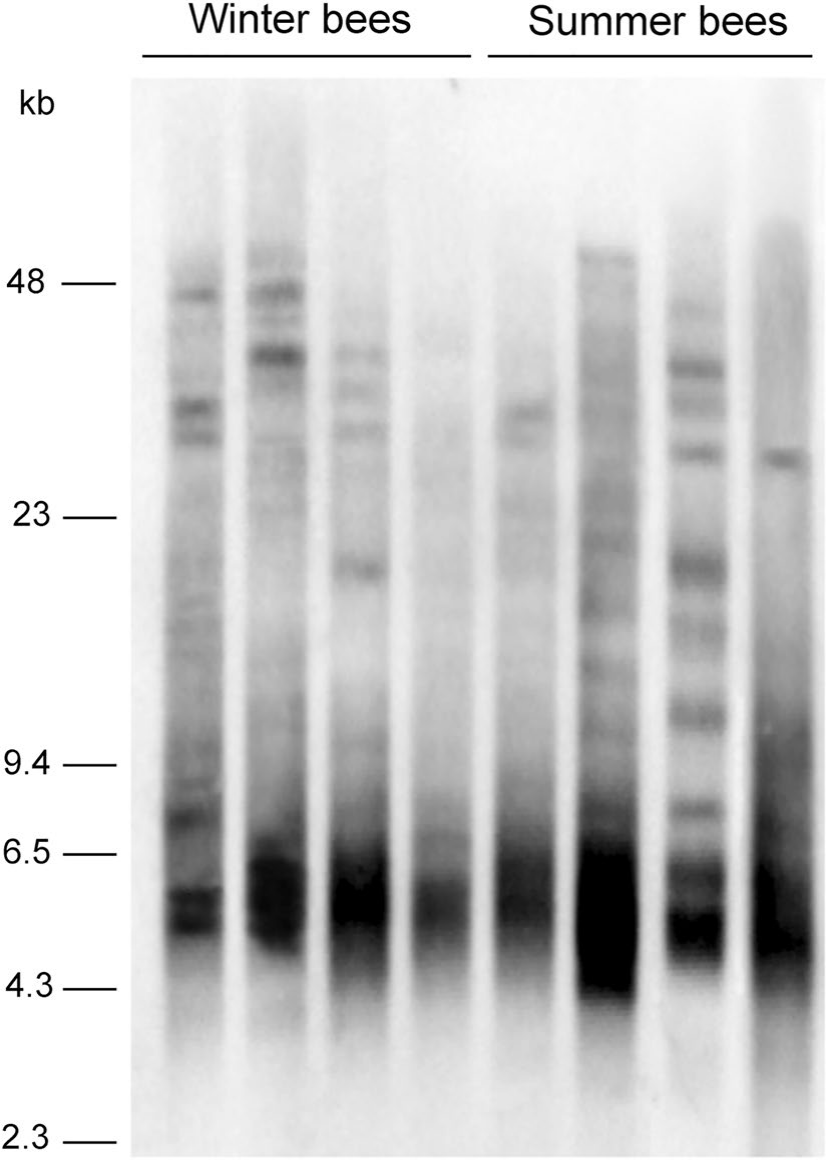
The TRF analysis. The TRF analysis was used to measure telomere length in the fat bodies of bees collected during winter (December-January) and summer (June-July). The DNA samples were digested with RsaI/Hinf I mixture, resolved on 1% agarose gel using pulsed field electrophoresis and visualized using Southern hybridization.

Using TRF analysis we evaluated telomere length in the fat body of winter and summer bees. Hybridization signals consisted of smears with numerous bands ranging from 2.3 kb to more than 48 kb, and the majority of the signal was between 4.3 – 6.5 kb. The intensity and position of the signals varied between individual samples and showed no consistent differences between the tested groups (Fig. 4).

### Seasonal changes in protein and carbohydrate levels in the fat body

**Figure 5:**
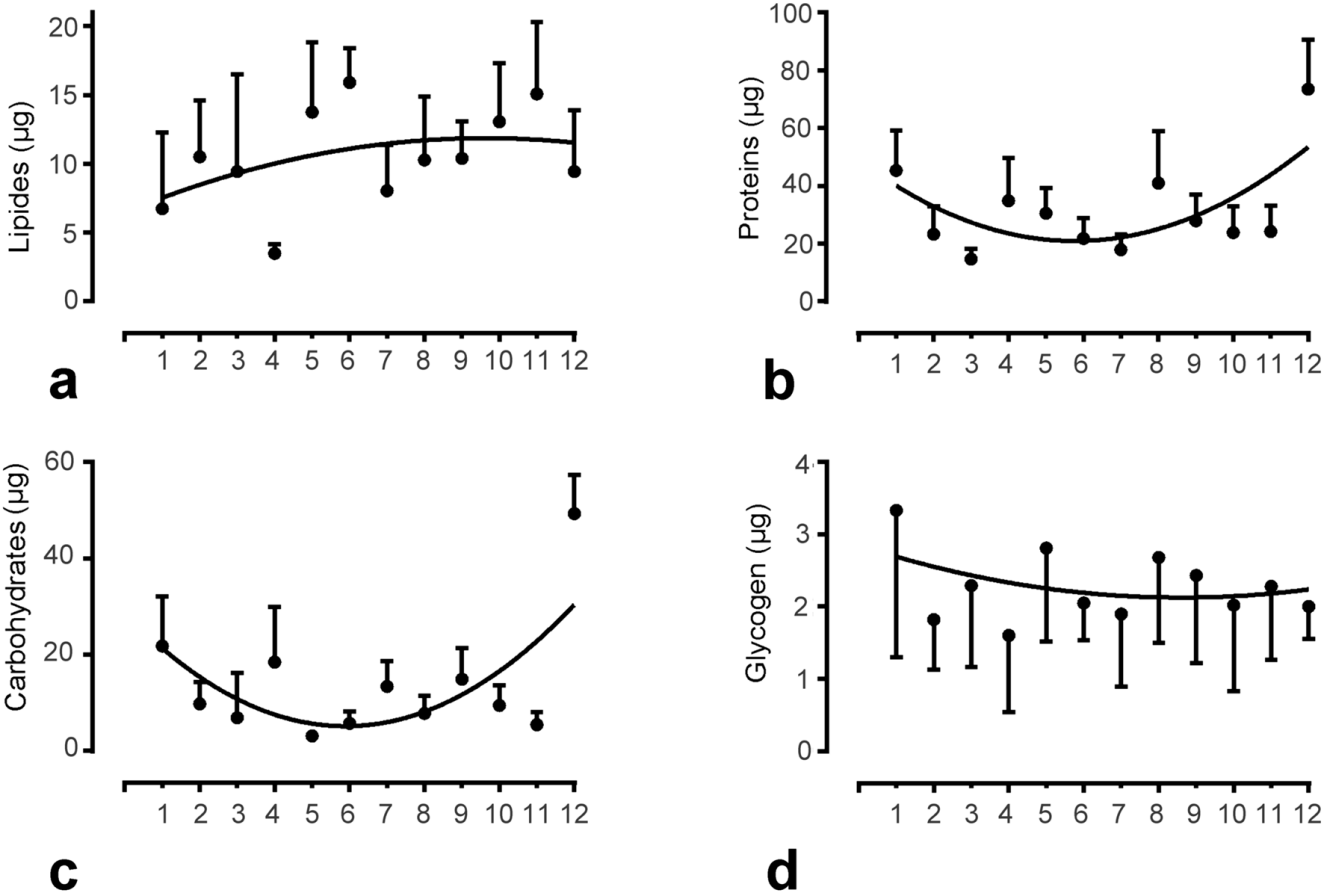
Energy reserves in the fat body. The total amounts of lipid **(a)**, protein **(b)**, carbohydrate **(c)**, and glycogen **(d)** in workers collected over 1 year. In March–October, experiments were performed on foragers of random age, and during the cold season (November–February) bees were sampled by random collection inside hives. Statistical significance was determined using one-way ANOVA and Tukey’s post-hoc tests. For statistical details, see Table S1 in supplements. Bars in graphs represent the mean ± SD.

Next, we explored how the seasonal changes in the fat body impact the basic nutrient content of the fat body. Therefore, we quantified lipids, proteins, free carbohydrates, and glycogen. As we assumed that the change in fat body mass was due to a change in cell size rather than cell number, we primarily focused on the total amount of nutrients per fat body of a bee, instead of quantifying the nutrient concentration (per mg of fat body). Compared to samples collected in summer, samples collected in December and January had significantly higher protein and carbohydrate contents; however, no clear trend was observed for lipids or glycogen reserves (Fig. 5, Table S1). In agreement with the presumption of a cell size increase in the winter season, when we calculated the nutrient concentrations (µg per mg of fat body), lipid and glycogen concentrations were highest in summer, which was not the case for carbohydrates and proteins (Fig. S2, Table S2).

### Seasonal changes in Vg levels in the hemolymph and fat body

**Figure 6:**
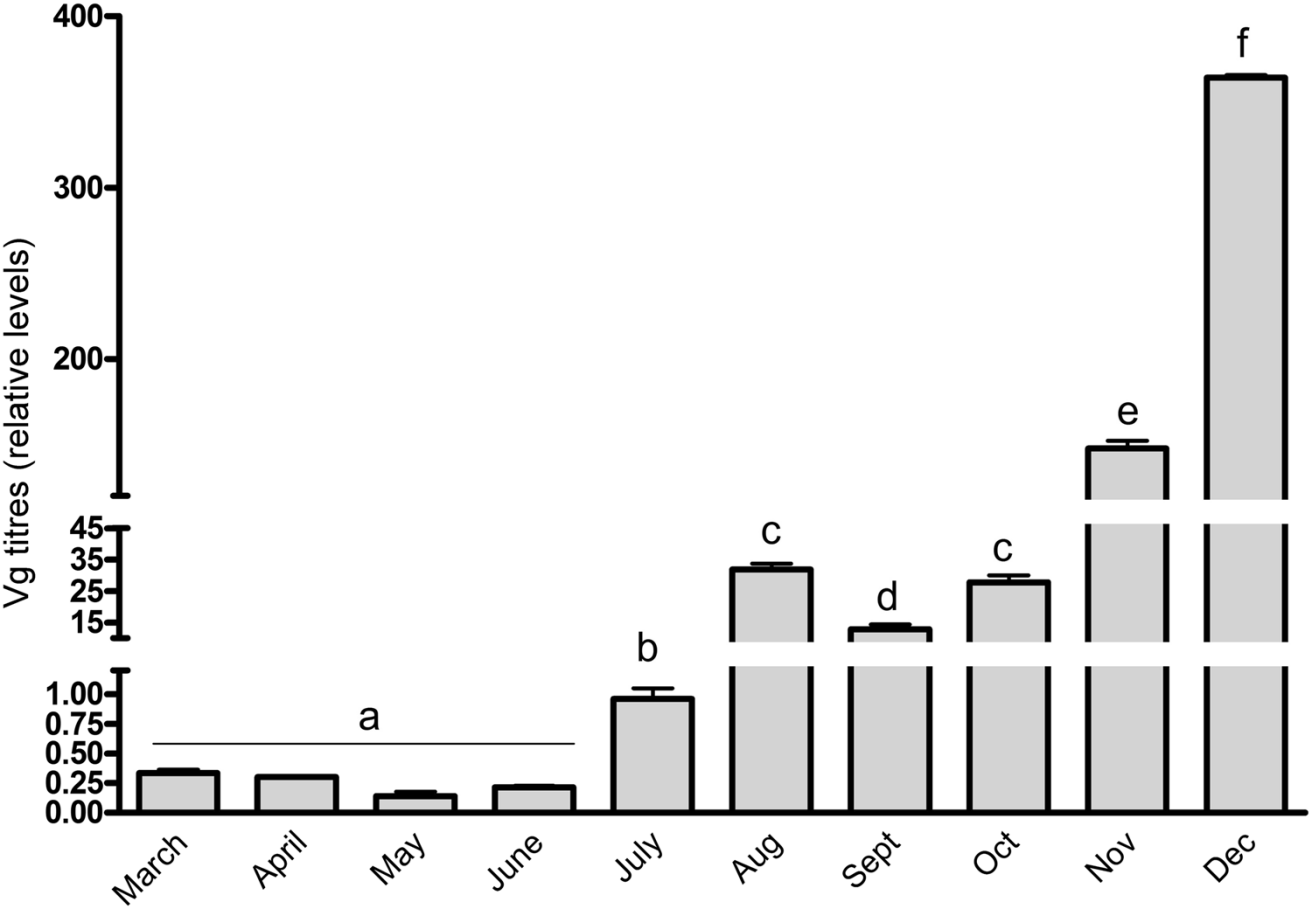
The vitellogenin titers in hemolymph. Vitellogenin (Vg) levels in the hemolymph of collected workers were evaluated using ELISA. In March–October, the experiments were performed on foragers of random age, and during the cold season (November–February) bees were sampled by random collection inside hives. Statistical significance was determined using one-way ANOVA and Tukey’s post-hoc tests (*P* < 0.05, indicated by different letters above the columns); n = 10. Bars in graphs represent the mean ± SD.

It is well known that Vg is synthesized in the fat body and then transported to the hemolymph, and it is also known that Vg levels are higher in winter bees^30^. To explore the change in Vg levels in greater detail and concurrently investigate how this trend correlates with the season-related changes in the fat bodies observed in our previous experiments, we assessed Vg levels in the hemolymph and fat bodies of bees collected at 1-month intervals from March to December. Although Vg levels in the fat bodes showed no significant seasonal differences **(**Fig. S3), the levels in hemolymph showed gradual seasonal trends throughout the year, with the peak during the cold season and the minimum in summer, and the overall difference was roughly 1,500-fold (*P* < 0.001; Fig. 6).

## Discussion

In adult somatic cells, which have high proliferative potential, telomerase activity is one of the factors related to regeneration capacity, thus promoting the health and lifespan of the organism. This study provides data supporting the idea that long-lived winter honeybee workers have higher telomerase activity in their fat bodies, which is presumably associated with a higher rate of DNA synthesis and increased fat body cell size and fat body mass. This is accompanied by higher levels of total protein and carbohydrate in the fat bodies in December and January, but surprisingly without significant trend in the lipid or glycogen contents.

The insect fat body, which is a major energy storage organ, produces a considerable amount of energy that is stored by trophocytes. It possesses a high metabolic rate and plays a crucial role in diapause survival and during unfavorable seasons, such as temperate winters^31^. To maximize energy production and storage, fat body cells are polyploid, with multiple DNA endoreduplication cycles^32^. Thus, the observed EdU signals in the fat body cells are a sign of ongoing endoreduplications rather than DNA replication connected to cell proliferation. Therefore, we speculate that the upregulation of telomerase activity and DNA replication and the increased size of the fat body cells and their nuclei in the winter season is a reflection of increased metabolic activity under low external temperatures. Based on our data, it seems that during winter, bees use their energy reserves deposited in the fat bodies to meet the ongoing energy demands but are able to refill these reserves. Surprisingly, no data have been published on the seasonal variations in the nutrient contents of the fat body of workers throughout the year. We did not observe differences in telomere length between summer and winter bees, which might suggest that telomere losses in the replicating DNA of the fat body cells are well balanced by upregulated telomerase activity. On the other hand, it needs to be pointed out that no differences in telomere length were observed in relation to the developmental stage, adult age or social status of honeybees^29^. The TRF signals in honeybees tend to consist of multiple bands that highly varied between individual bees, and based on this variation it might be difficult to see a real extent of telomere lengthening/shortening.

In our previous study, we observed upregulated telomerase activity and high-level DNA synthesis in the fat bodies of pre-diapause queens of the bumblebee *Bombus terrestris*. This finding was explained as a way to meet the requirement for enhanced metabolic activity to build up sufficient energy reserves and increase fat body mass before diapause^33^ and is consistent with the fact that *B. terrestris* queens are not able to refill energy reserves during diapause. This illustrates the difference in the way in which *B. terrestris* and *A. mellifera* survive temperate winters.

We did not detect DNA synthesis in the heads of worker bees; thus, the reason for the presence of telomerase activity in these tissues remains unclear. We speculate that the rate of DNA synthesis may be too low to detect or that the observed telomerase activity may be due to some non-telomere-related functions of telomerase. For instance, it is known that telomeres and telomerase are also related to the biogenesis and function of the mitochondria and the production of reactive oxygen species^34–36^. Similarly, there has been speculation about the role of elevated Vg levels in the lifespan extension of winter bees. Vg is synthesized in the fat body and accumulates in hemolymph, and its levels are negatively impacted by brood rearing and even by exposure to brood pheromone alone^30^. In addition to its role as a precursor of yolk protein in reproduction, Vg has also been implicated in the defense against reactive oxygen species^37–39^ and as an enhancer of innate immunity^40^.

The important findings of this study are the seasonal fluctuations in several tested parameters, including fat body size, telomerase activity, and Vg levels, which are consistent with the previously reported progressive seasonal changes in the JH titers of workers^11^. JH is a key player in endocrine signaling, and in bees, it is associated with caste determination and the regulation of division of labor^41^. In most insects, JH and Vg titers are positively correlated. In contrast, honeybees show an inhibitory effect of Vg on JH titer, and JH and Vg titers show opposite patterns^7–9,42^. Consistent with the finding that JH accelerates aging in insects^43^, JH titers are low in nurse bees and high in foragers, and an elevation in JH levels, caused by a shortage of foragers in a colony or treatment of bees with JH, accelerated bee maturation and led to precocious foraging^44^. In workers, the JH titer fluctuates over the course of a year, with a peak in summer and a nadir in winter^11^. However, the winter decline in JH levels could be mimicked in summer by experimentally reducing the temperature, suggesting that the seasonal changes in JH titers are not associated with photoperiod, but rather with temperature and temperature-correlated brooding activity changes^11,45^. In contrast, the change in fat body mass seems to be stimulated by a shortened photoperiod^46,47^.

Telomerase is commonly considered to be a lifespan-regulating factor, and the elevated levels in honeybee fat bodies, together with the decline in JH and the increase in Vg levels and fat body mass, are associated with the winter generation of honeybee workers. However, we hypothesize that the levels of these factors are not set in the bees that hatch at the end of summer or in early autumn as a long-lasting winter bee generation, but vary over the course of the year, depending on the interplay of external factors, such as photoperiod, temperature, and brood rearing. Considering all these observations, we suggest that photoperiod shortening stimulates DNA replication in fat body cells, which increases the metabolic activity in the cells and Vg synthesis. Although JH levels are primarily set by temperature-dependent brooding activity, increased levels of Vg decrease JH levels, potentially leading to a prolonged lifespan.

## Materials and methods

### Bees

The experiments were conducted with the honey bee *Apis mellifera carnica*. Bees were kept in the apiary of the Biology Centre in České Budějovice (48°58′31.924"N, 14°26′44.671″E; 390 m), and five colonies were used for experiments. The bees were maintained according to standard bee-keeping techniques. From each colony, nurse or forager bees were collected from March to October, and over the course of cold season (November—February) bees were sampled by a random collection inside hives. All experiments with bees were performed in accordance with relevant guidelines and regulations.

### Evaluation of telomerase activity

We homogenized the dissected tissue in 4 volumes of extraction buffer (10 mM Tris/HCl, pH 7.6; 1 mM EGTA; 1 mM MgCl_2_; 0.1 mM benzamidine (PMSF); 10% (v/v) glycerol; 0.5% (w/v) CHAPS; 5 mM 2-mercaptoethanol, and 40 U/ml RNase inhibitor (Promega). The homogenized samples were chilled on ice for 30 min, centrifuged (12,000 g; 20 min; 4 °C), and supernatants were collected and stored at -80 °C for further use. Total protein concentration was specified using a BCA protein assay reagent kit (Thermo Fisher Scientific).

Telomerase activity was defined using a Telomerase repeat amplification protocol (TRAP) assay combined with quantitative Real-time PCR as described formerly^28^ The extension products were amplified by PCR with TS primer and Bm-CXa primer (5′-GTGTAACCTAACCTAACC-3′). Each reaction was performed in 25 µl total volume and contained 12.5 µl Xceed qPCR SG 2 × Mix (Institute of Applied Biotechnologies), 5 ng of protein and 5 pmol of both TS and Bm-CXa primer. Samples were incubated at 30 °C for 60 min, and PCR was performed with 30 cycles at 94 °C for 30 s and 60 °C for 30 s. Telomerase activity quantitation was achieved using a Light Cycler CFX96 BioRad Real-time PCR system (Bio-Rad Laboratories). Negative controls for tested samples were prepared by incubation of protein extracts with 0.5 µl of 1 µg/µl RNase A (Sigma-Aldrich) for 20 min at 37 °C performed prior to TS primer elongation. To visualize the TRAP products, the TRAP products were end-labeled with [γ-32P]dATP using T4 polynucleotide kinase, resolved on 12% polyacrylamide gels and visualized on Typhoon PhosphorImager scanner system.

### Cloning and sequencing of TRAP products

We purified the TRAP products with NucleoSpin Gel and PCR Clean-up (Machery-Nagel) and cloned into pGEM-T easy vector (Promega Corporation). Plasmid DNA was isolated using Nucleospin Plasmid Quickpure Kit (Macherey–Nagel) and the inserts were sequenced using ABI PRISM 3.1 (Applied Biosystems) using T7 and SP6 primers.

### Detection of DNA replication in fat bodies

We used Click-iT EdU Alexa Fluoor 488 Imaging Kit (Invitrogen) for location of S-phase nuclei in the fat bodies. The fat bodies were dissected and cultivated for 24 h in M3 Insect Medium (Sigma-Aldrich) supplemented with fetal bovine serum (12%), 0.4 mM EdU (5-ethynyl-2′deoxyuridine), insulin (0. 1%), and penicillin/streptomycin solution (1%). We fixed samples in 3.7% formaldehyde in phosphate-buffered saline (PBS) for 2 h and washed in PBS (three times for 15 min each) and PBS, 0.5% v/v Triton X-100 for 15 min. Then we incubated the samples with 3% w/v bovine serum albumin in PBS for 30 min and then in Click- iT reaction cocktail for 12 h at 4 °C. After washing in PBS (three times for 15 min), the samples were incubated in a solution of 4′-6-diamidino-2-phenylindole (DAPI, 1 µg DAPI in 1 ml distilled H_2_O) and washed in 1 ml distilled H_2_O (three times for 15 min). Finally, samples were dehydrated in ethanol (50, 70, 90, and 100%) and mounted on microscope slides in methyl-salicylate (Sigma-Aldrich). Samples were observed, and images recorded under a confocal microscope (Olympus FV-1000).

### Cell and nuclear sizes in fat body

The dissected fat bodies were fixed in 3.7% formaldehyde in PBS for one hour, washed in PBS (three times for 15 min each), in PBS, 0.5% v/v Triton X-100 for 15 min, and in 1 ml distilled H_2_O (three times for 15 min). Two sets of staining techniques were performed. First, samples were incubated in a solution of DAPI (1 µg/1 ml distilled H_2_O), dehydrated in ethanol (50, 70, 90, and 100%), and mounted on microscope slides in methyl-salicylate (Sigma-Aldrich). Samples were observed, and images recorded under a confocal microscope (Olympus FV-1000). Second, samples were stained with Oil Red IV (Carl Roth) and hematoxylin (H&E Staining Kit, Carl Roth). Samples were incubated in 60% isopropanol, stained in freshly prepared working solution of Oil Red IV for 25 min, and thoroughly washed in distilled water. Oil Red IV stock solution was prepared by dissolving 0.5 g in 50 ml isopropanol (1% w/v) and stored at room temperature. The working solution was prepared by diluting the stock solution 2:3 with distilled water and filtered immediately before use. Samples were incubated in H&E solution 1(H&E Staining Kit, Carl Roth) for 1 min, thoroughly washed in distilled water, incubated in 0.1% HCl for 15 s, and thoroughly washed in distilled water. Samples were incubated in distilled water for 6 min and mounted on microscope slides in glycerol/TBS (2:3) mounting medium. Samples were visualized using a light microscope (Motic) and evaluated by Adobe Photoshop.

### Telomere restriction fragment (TRF) analysis

The TRF analysis was used to measure telomere length in the fat bodies of winter and summer bees. Genomic DNAs were extracted from bees collected during June and July and bees collected during December and January. To extract DNA, the standard phenol–chloroform-isoamyl alcohol procedure was used. The quality of the DNA samples was checked on 1% agarose gel electrophoresis with ethidium bromide staining. DNA samples (1 µg) were digested with Rsa I and Hinf I (New Englands Biolabs) and resolved on 1% agarose gel using pulsed field electrophoresis (Bio-Rad). DNA was blotted onto a positively charged nylon membrane (Hybond-N + ; GE Healthcare), hybridized with a DIG-labeled telomere probe, and detected using chemiluminescence as previously described^29^. The TRF analysis was done in two independent experiments; each experiment included four samples per the season.

### Determination of nutrients

Fat bodies were dissected from experimental individuals in Ringer solution under the dissecting microscope and weighted. Levels of nutrients were evaluated in fat bodies as described previously^48,49^. Briefly, total lipids were extracted by chloroform:methanol (2:1) mixture and quantified using the sulfo-phospho-vanillin method. The optical densities at 546 nm were measured spectrophotometrically and were converted to µg of lipids using a calibration curve with specified doses of oleic acid. For protein determination, fat bodies were homogenized in 0.2 M Tris–HCl buffer (pH 7.8), centrifuged, and the supernatants were used for protein quantification with the Bicinchoninic Acid Protein Assay Kit (Sigma-Aldrich). The optical densities of tested samples were measured at 562 nm and converted to µg of proteins using the bovine serum albumin standard curve. The anthrone method was used to determine carbohydrate and glycogen levels. Fat bodies were homogenized in 70% ethanol. The extracts were evaporated and used for free carbohydrate determination, and sediments were used for glycogen determination. The optical densities were measured at 620 nm and expressed in equivalents of glucose standard.

### Vitellogenin quantification

Vitellogenin levels in the hemolymph of collected workers were evaluated using a direct ELISA, and we used the polyclonal rabbit antibody raised against vitellogenin purified by electrophoretic methods from the queen hemolymph. The 96-well High Binding ELISA microplates (Corning Inc.) were precoated with a sample of 0.002 μl hemolymph equivalent overnight, blocked with 3% BSA (bovine serum albumin) and incubated with an anti-vitellogenin antibody (1:20,000 dilution) and a swine anti-rabbit IgG HRP as second antibody (1:2,000; LabNed.com). The reaction was visualized by the ELISA substrate 3,3′5,5′-tetramethylbenzidine, and the absorbance values were determined in a microplate reader at 450 nm. The results were expressed as relative absorbance normalised per 1 µl of hemolymph.

### Statistical analysis

GraphPad Prism 6.0 (GraphPad Software, San Diego, CA, USA) was used for one-way ANOVA tests followed by Tukey’s multiple comparison test. Number of replicates is specified in the figure legends, and the bars in graphs represent the mean ± SD.

### Image processing

All images were processed with Adobe Photoshop CS6 (Adobe Systems; V.6.0.1), using proportionate adjustments of brightness or contrast.

## Supplementary information

Supplementary Information.
