# Supplementary material for: Seasonality in telomerase activity in relation to cell size, DNA replication, and nutrients in the fat body of Apis mellifera
Source: Sci Rep. 2021 Jan 12;11:592. doi: 10.1038/s41598-020-79912-9 (PMC7803764; doi:10.1038/s41598-020-79912-9)
Supplement: Supplementary file 1 — Supplementary Information. [file 41598_2020_79912_MOESM1_ESM.docx]

**Seasonality in telomerase activity in relation to cell size, DNA replication, and nutrients in the fat body of *Apis mellifera***

Justina Koubová^1,2,§^, Michala Sábová^1,§^, Miloslav Brejcha^1,2^, Dalibor Kodrík^1,2^, and Radmila Čapková Frydrychová^1,2^*

^1^ Biology Centre of the Czech Academy of Sciences, Institute of Entomology, Branišovská 31, 370 05 České Budějovice, Czech Republic; Radmila.Frydrychova@hotmail.com

^2^ Faculty of Science, University of South Bohemia, České Budějovice, Czech Republic

§ Both authors contributed equally to this work.

* Corresponding author

**Supplementary material Figure S1.**

**
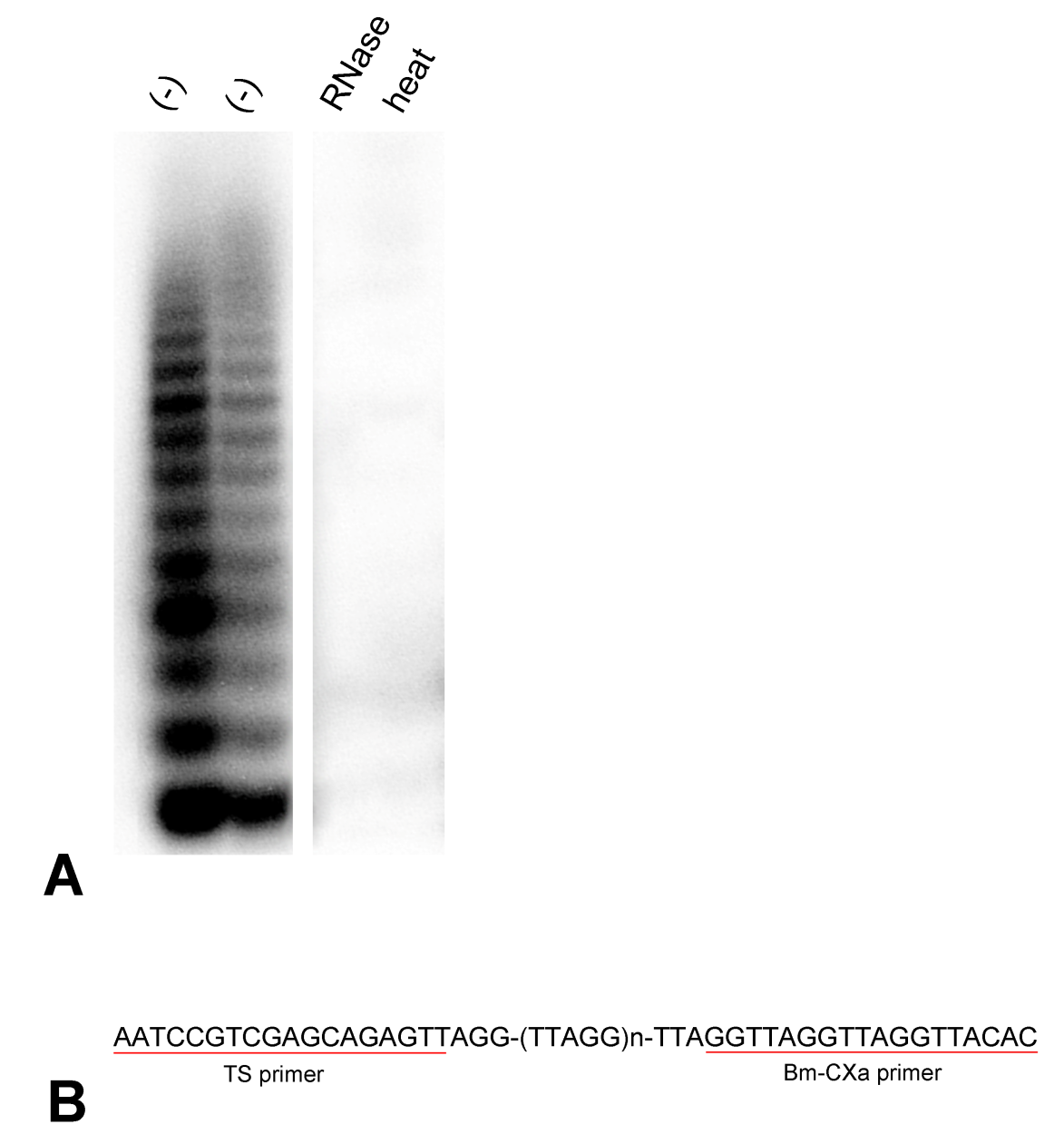
**

**Fig. S1** The specificity of TRAP assay. **(a)** The TRAP amplification products from ovaries (extracted from late pupae) and heads of adult workers were end-labeled with [γ-32P]dATP using T4 polynucleotide kinase, resolved on 12% polyacrylamide gels and visualized on Typhoon PhosphorImager scanner system. The telomerase activity was manifested by the presence of a characteristic ladder pattern. No telomerase activity was detected in negative controls after RNase or heat treatment. **(b)** The TRAP amplification products were cloned into pGEM-T easy vector, and the cloned inserts were sequenced with T7 and SP6 primers. The sequencing revealed a variable number of TTAGG repeats placed between the TS and Bm-CXa primers. Collectively, six samples representing heads, fat bodies and ovaries (as a positive control) were sequenced. The number of TTAGG repeats in the sequenced clones varied from three to eight.

**Supplementary material Figure S2.**





**Fig. S2** Nutrient concentrations in the fat body. Concentrations of lipid **(a)**, protein **(b)**, carbohydrate **(c)**, and glycogen **(d)** in workers collected over 1 year. In March–October, experiments were performed on foragers of random age, and during the cold season (November–February) bees were sampled by random collection inside hives. Statistical significance was determined using one-way ANOVA and Tukey’s post-hoc tests. Bars in graphs represent the mean ± SD.

**Supplementary material Figure S3.**


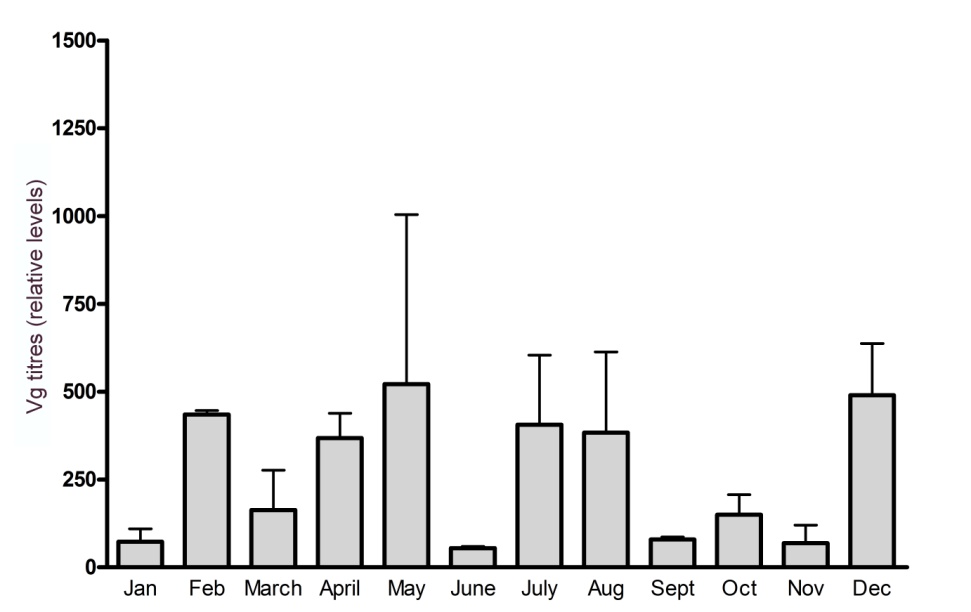


**Fig. S3** The vitellogenin titers in fat bodies. Vitellogenin (Vg) levels in fat bodies of collected workers were evaluated using ELISA. In March–October, the experiments were performed on foragers of random age, and during the cold season (November–February) bees were sampled by random collection inside hives. Bars in graphs represent the mean ± SD; n = 3.

**Supplementary material Table S1.**

Statistical details for Figure 4 (nutrient amounts). ns, not significant; * p<0.05; ** p<0.01; *** p<0.0001; **** p<0.00001

Figure 4A - lipids: One-way ANOVA. Tukey’s post test.

| **Tukey's multiple comparisons test** | **Mean diff.** | **95% CI of diff.** | **Significance** |
| --- | --- | --- | --- |
| 1 vs. 2 | -3.760 | -11.48 to 3.962 | ns |
| 1 vs. 3 | -2.700 | -10.42 to 5.022 | ns |
| 1 vs. 4 | 3.250 | -5.091 to 11.59 | ns |
| 1 vs. 5 | -7.020 | -14.74 to 0.7018 | ns |
| 1 vs. 6 | -9.170 | -17.51 to -0.8295 | * |
| 1 vs. 7 | -1.300 | -9.641 to 7.041 | ns |
| 1 vs. 8 | -3.540 | -11.26 to 4.182 | ns |
| 1 vs. 9 | -3.650 | -11.99 to 4.691 | ns |
| 1 vs. 10 | -6.310 | -14.65 to 2.031 | ns |
| 1 vs. 11 | -8.340 | -16.68 to 0.0005354 | ns |
| 1 vs. 12 | -2.710 | -11.05 to 5.631 | ns |
| 2 vs. 3 | 1.060 | -6.662 to 8.782 | ns |
| 2 vs. 4 | 7.010 | -1.331 to 15.35 | ns |
| 2 vs. 5 | -3.260 | -10.98 to 4.462 | ns |
| 2 vs. 6 | -5.410 | -13.75 to 2.931 | ns |
| 2 vs. 7 | 2.460 | -5.881 to 10.80 | ns |
| 2 vs. 8 | 0.2200 | -7.502 to 7.942 | ns |
| 2 vs. 9 | 0.1100 | -8.231 to 8.451 | ns |
| 2 vs. 10 | -2.550 | -10.89 to 5.791 | ns |
| 2 vs. 11 | -4.580 | -12.92 to 3.761 | ns |
| 2 vs. 12 | 1.050 | -7.291 to 9.391 | ns |
| 3 vs. 4 | 5.950 | -2.391 to 14.29 | ns |
| 3 vs. 5 | -4.320 | -12.04 to 3.402 | ns |
| 3 vs. 6 | -6.470 | -14.81 to 1.871 | ns |
| 3 vs. 7 | 1.400 | -6.941 to 9.741 | ns |
| 3 vs. 8 | -0.8400 | -8.562 to 6.882 | ns |
| 3 vs. 9 | -0.9500 | -9.291 to 7.391 | ns |
| 3 vs. 10 | -3.610 | -11.95 to 4.731 | ns |
| 3 vs. 11 | -5.640 | -13.98 to 2.701 | ns |
| 3 vs. 12 | -0.01000 | -8.351 to 8.331 | ns |
| 4 vs. 5 | -10.27 | -18.61 to -1.929 | ** |
| 4 vs. 6 | -12.42 | -21.34 to -3.504 | *** |
| 4 vs. 7 | -4.550 | -13.47 to 4.366 | ns |
| 4 vs. 8 | -6.790 | -15.13 to 1.551 | ns |
| 4 vs. 9 | -6.900 | -15.82 to 2.016 | ns |
| 4 vs. 10 | -9.560 | -18.48 to -0.6436 | * |
| 4 vs. 11 | -11.59 | -20.51 to -2.674 | ** |
| 4 vs. 12 | -5.960 | -14.88 to 2.956 | ns |
| 5 vs. 6 | -2.150 | -10.49 to 6.191 | ns |
| 5 vs. 7 | 5.720 | -2.621 to 14.06 | ns |
| 5 vs. 8 | 3.480 | -4.242 to 11.20 | ns |
| 5 vs. 9 | 3.370 | -4.971 to 11.71 | ns |
| 5 vs. 10 | 0.7100 | -7.631 to 9.051 | ns |
| 5 vs. 11 | -1.320 | -9.661 to 7.021 | ns |
| 5 vs. 12 | 4.310 | -4.031 to 12.65 | ns |
| 6 vs. 7 | 7.870 | -1.046 to 16.79 | ns |
| 6 vs. 8 | 5.630 | -2.711 to 13.97 | ns |
| 6 vs. 9 | 5.520 | -3.396 to 14.44 | ns |
| 6 vs. 10 | 2.860 | -6.056 to 11.78 | ns |
| 6 vs. 11 | 0.8300 | -8.086 to 9.746 | ns |
| 6 vs. 12 | 6.460 | -2.456 to 15.38 | ns |
| 7 vs. 8 | -2.240 | -10.58 to 6.101 | ns |
| 7 vs. 9 | -2.350 | -11.27 to 6.566 | ns |
| 7 vs. 10 | -5.010 | -13.93 to 3.906 | ns |
| 7 vs. 11 | -7.040 | -15.96 to 1.876 | ns |
| 7 vs. 12 | -1.410 | -10.33 to 7.506 | ns |
| 8 vs. 9 | -0.1100 | -8.451 to 8.231 | ns |
| 8 vs. 10 | -2.770 | -11.11 to 5.571 | ns |
| 8 vs. 11 | -4.800 | -13.14 to 3.541 | ns |
| 8 vs. 12 | 0.8300 | -7.511 to 9.171 | ns |
| 9 vs. 10 | -2.660 | -11.58 to 6.256 | ns |
| 9 vs. 11 | -4.690 | -13.61 to 4.226 | ns |
| 9 vs. 12 | 0.9400 | -7.976 to 9.856 | ns |
| 10 vs. 11 | -2.030 | -10.95 to 6.886 | ns |
| 10 vs. 12 | 3.600 | -5.316 to 12.52 | ns |
| 11 vs. 12 | 5.630 | -3.286 to 14.55 | ns |

Figure 4B - proteins: One-way ANOVA. Tukey’s post test.

| **Tukey's multiple comparisons test** | **Mean diff.** | **95% CI of diff.** | **Significance** |
| --- | --- | --- | --- |
| 1 vs. 2 | 21.96 | 3.538 to 40.38 | ** |
| 1 vs. 3 | 30.64 | 10.74 to 50.54 | **** |
| 1 vs. 4 | 10.40 | -9.498 to 30.30 | ns |
| 1 vs. 5 | 14.76 | -2.716 to 32.24 | ns |
| 1 vs. 6 | 23.48 | 5.058 to 41.90 | ** |
| 1 vs. 7 | 27.36 | 9.884 to 44.84 | **** |
| 1 vs. 8 | 4.280 | -15.62 to 24.18 | ns |
| 1 vs. 9 | 17.36 | -1.062 to 35.78 | ns |
| 1 vs. 10 | 21.44 | 3.018 to 39.86 | ** |
| 1 vs. 11 | 21.08 | 2.658 to 39.50 | * |
| 1 vs. 12 | -28.16 | -46.58 to -9.738 | *** |
| 2 vs. 3 | 8.680 | -11.22 to 28.58 | ns |
| 2 vs. 4 | -11.56 | -31.46 to 8.338 | ns |
| 2 vs. 5 | -7.200 | -24.68 to 10.28 | ns |
| 2 vs. 6 | 1.520 | -16.90 to 19.94 | ns |
| 2 vs. 7 | 5.400 | -12.08 to 22.88 | ns |
| 2 vs. 8 | -17.68 | -37.58 to 2.218 | ns |
| 2 vs. 9 | -4.600 | -23.02 to 13.82 | ns |
| 2 vs. 10 | -0.5200 | -18.94 to 17.90 | ns |
| 2 vs. 11 | -0.8800 | -19.30 to 17.54 | ns |
| 2 vs. 12 | -50.12 | -68.54 to -31.70 | **** |
| 3 vs. 4 | -20.24 | -41.51 to 1.031 | ns |
| 3 vs. 5 | -15.88 | -34.91 to 3.146 | ns |
| 3 vs. 6 | -7.160 | -27.06 to 12.74 | ns |
| 3 vs. 7 | -3.280 | -22.31 to 15.75 | ns |
| 3 vs. 8 | -26.36 | -47.63 to -5.089 | ** |
| 3 vs. 9 | -13.28 | -33.18 to 6.618 | ns |
| 3 vs. 10 | -9.200 | -29.10 to 10.70 | ns |
| 3 vs. 11 | -9.560 | -29.46 to 10.34 | ns |
| 3 vs. 12 | -58.80 | -78.70 to -38.90 | **** |
| 4 vs. 5 | 4.360 | -14.67 to 23.39 | ns |
| 4 vs. 6 | 13.08 | -6.818 to 32.98 | ns |
| 4 vs. 7 | 16.96 | -2.066 to 35.99 | ns |
| 4 vs. 8 | -6.120 | -27.39 to 15.15 | ns |
| 4 vs. 9 | 6.960 | -12.94 to 26.86 | ns |
| 4 vs. 10 | 11.04 | -8.858 to 30.94 | ns |
| 4 vs. 11 | 10.68 | -9.218 to 30.58 | ns |
| 4 vs. 12 | -38.56 | -58.46 to -18.66 | **** |
| 5 vs. 6 | 8.720 | -8.756 to 26.20 | ns |
| 5 vs. 7 | 12.60 | -3.877 to 29.08 | ns |
| 5 vs. 8 | -10.48 | -29.51 to 8.546 | ns |
| 5 vs. 9 | 2.600 | -14.88 to 20.08 | ns |
| 5 vs. 10 | 6.680 | -10.80 to 24.16 | ns |
| 5 vs. 11 | 6.320 | -11.16 to 23.80 | ns |
| 5 vs. 12 | -42.92 | -60.40 to -25.44 | **** |
| 6 vs. 7 | 3.880 | -13.60 to 21.36 | ns |
| 6 vs. 8 | -19.20 | -39.10 to 0.6976 | ns |
| 6 vs. 9 | -6.120 | -24.54 to 12.30 | ns |
| 6 vs. 10 | -2.040 | -20.46 to 16.38 | ns |
| 6 vs. 11 | -2.400 | -20.82 to 16.02 | ns |
| 6 vs. 12 | -51.64 | -70.06 to -33.22 | **** |
| 7 vs. 8 | -23.08 | -42.11 to -4.054 | ** |
| 7 vs. 9 | -10.00 | -27.48 to 7.476 | ns |
| 7 vs. 10 | -5.920 | -23.40 to 11.56 | ns |
| 7 vs. 11 | -6.280 | -23.76 to 11.20 | ns |
| 7 vs. 12 | -55.52 | -73.00 to -38.04 | **** |
| 8 vs. 9 | 13.08 | -6.818 to 32.98 | ns |
| 8 vs. 10 | 17.16 | -2.738 to 37.06 | ns |
| 8 vs. 11 | 16.80 | -3.098 to 36.70 | ns |
| 8 vs. 12 | -32.44 | -52.34 to -12.54 | **** |
| 9 vs. 10 | 4.080 | -14.34 to 22.50 | ns |
| 9 vs. 11 | 3.720 | -14.70 to 22.14 | ns |
| 9 vs. 12 | -45.52 | -63.94 to -27.10 | **** |
| 10 vs. 11 | -0.3600 | -18.78 to 18.06 | ns |
| 10 vs. 12 | -49.60 | -68.02 to -31.18 | **** |
| 11 vs. 12 | -49.24 | -67.66 to -30.82 | **** |

Figure 4C – carbohydrates: One-way ANOVA. Tukey’s post test.

| **Tukey's multiple comparisons test** | **Mean diff.** | **95% CI of diff.** | **Significance** |
| --- | --- | --- | --- |
| 1 vs. 2 | 12.02 | 1.094 to 22.95 | * |
| 1 vs. 3 | 14.87 | 2.413 to 27.33 | ** |
| 1 vs. 4 | 3.320 | -7.606 to 14.25 | ns |
| 1 vs. 5 | 18.65 | 7.724 to 29.58 | **** |
| 1 vs. 6 | 16.07 | 5.144 to 27.00 | *** |
| 1 vs. 7 | 8.390 | -3.411 to 20.19 | ns |
| 1 vs. 8 | 13.97 | 2.169 to 25.77 | ** |
| 1 vs. 9 | 6.890 | -4.036 to 17.82 | ns |
| 1 vs. 10 | 12.35 | 1.424 to 23.28 | * |
| 1 vs. 11 | 16.34 | 5.975 to 26.71 | **** |
| 1 vs. 12 | -27.52 | -38.45 to -16.59 | **** |
| 2 vs. 3 | 2.850 | -9.607 to 15.31 | ns |
| 2 vs. 4 | -8.700 | -19.63 to 2.226 | ns |
| 2 vs. 5 | 6.630 | -4.296 to 17.56 | ns |
| 2 vs. 6 | 4.050 | -6.876 to 14.98 | ns |
| 2 vs. 7 | -3.630 | -15.43 to 8.171 | ns |
| 2 vs. 8 | 1.950 | -9.851 to 13.75 | ns |
| 2 vs. 9 | -5.130 | -16.06 to 5.796 | ns |
| 2 vs. 10 | 0.3300 | -10.60 to 11.26 | ns |
| 2 vs. 11 | 4.320 | -6.045 to 14.69 | ns |
| 2 vs. 12 | -39.54 | -50.47 to -28.61 | **** |
| 3 vs. 4 | -11.55 | -24.01 to 0.9075 | ns |
| 3 vs. 5 | 3.780 | -8.677 to 16.24 | ns |
| 3 vs. 6 | 1.200 | -11.26 to 13.66 | ns |
| 3 vs. 7 | -6.480 | -19.71 to 6.752 | ns |
| 3 vs. 8 | -0.9000 | -14.13 to 12.33 | ns |
| 3 vs. 9 | -7.980 | -20.44 to 4.477 | ns |
| 3 vs. 10 | -2.520 | -14.98 to 9.937 | ns |
| 3 vs. 11 | 1.470 | -10.50 to 13.44 | ns |
| 3 vs. 12 | -42.39 | -54.85 to -29.93 | **** |
| 4 vs. 5 | 15.33 | 4.404 to 26.26 | *** |
| 4 vs. 6 | 12.75 | 1.824 to 23.68 | ** |
| 4 vs. 7 | 5.070 | -6.731 to 16.87 | ns |
| 4 vs. 8 | 10.65 | -1.151 to 22.45 | ns |
| 4 vs. 9 | 3.570 | -7.356 to 14.50 | ns |
| 4 vs. 10 | 9.030 | -1.896 to 19.96 | ns |
| 4 vs. 11 | 13.02 | 2.655 to 23.39 | ** |
| 4 vs. 12 | -30.84 | -41.77 to -19.91 | **** |
| 5 vs. 6 | -2.580 | -13.51 to 8.346 | ns |
| 5 vs. 7 | -10.26 | -22.06 to 1.541 | ns |
| 5 vs. 8 | -4.680 | -16.48 to 7.121 | ns |
| 5 vs. 9 | -11.76 | -22.69 to -0.8341 | * |
| 5 vs. 10 | -6.300 | -17.23 to 4.626 | ns |
| 5 vs. 11 | -2.310 | -12.68 to 8.055 | ns |
| 5 vs. 12 | -46.17 | -57.10 to -35.24 | **** |
| 6 vs. 7 | -7.680 | -19.48 to 4.121 | ns |
| 6 vs. 8 | -2.100 | -13.90 to 9.701 | ns |
| 6 vs. 9 | -9.180 | -20.11 to 1.746 | ns |
| 6 vs. 10 | -3.720 | -14.65 to 7.206 | ns |
| 6 vs. 11 | 0.2700 | -10.10 to 10.64 | ns |
| 6 vs. 12 | -43.59 | -54.52 to -32.66 | **** |
| 7 vs. 8 | 5.580 | -7.036 to 18.20 | ns |
| 7 vs. 9 | -1.500 | -13.30 to 10.30 | ns |
| 7 vs. 10 | 3.960 | -7.841 to 15.76 | ns |
| 7 vs. 11 | 7.950 | -3.334 to 19.23 | ns |
| 7 vs. 12 | -35.91 | -47.71 to -24.11 | **** |
| 8 vs. 9 | -7.080 | -18.88 to 4.721 | ns |
| 8 vs. 10 | -1.620 | -13.42 to 10.18 | ns |
| 8 vs. 11 | 2.370 | -8.914 to 13.65 | ns |
| 8 vs. 12 | -41.49 | -53.29 to -29.69 | **** |
| 9 vs. 10 | 5.460 | -5.466 to 16.39 | ns |
| 9 vs. 11 | 9.450 | -0.9152 to 19.82 | ns |
| 9 vs. 12 | -34.41 | -45.34 to -23.48 | **** |
| 10 vs. 11 | 3.990 | -6.375 to 14.36 | ns |
| 10 vs. 12 | -39.87 | -50.80 to -28.94 | **** |
| 11 vs. 12 | -43.86 | -54.23 to -33.49 | **** |

Figure 4D - glycogen: One-way ANOVA. Tukey’s post test

| **Tukey's multiple comparisons test** | **Mean diff.** | **95% CI of diff.** | **Significance** |
| --- | --- | --- | --- |
| 1 vs. 2 | 1.510 | -0.3352 to 3.355 | ns |
| 1 vs. 3 | 1.040 | -0.9688 to 3.049 | ns |
| 1 vs. 4 | 1.730 | -0.1152 to 3.575 | ns |
| 1 vs. 5 | 0.5200 | -1.220 to 2.260 | ns |
| 1 vs. 6 | 1.280 | -0.5652 to 3.125 | ns |
| 1 vs. 7 | 1.430 | -0.3097 to 3.170 | ns |
| 1 vs. 8 | 0.6500 | -1.195 to 2.495 | ns |
| 1 vs. 9 | 0.9000 | -0.9452 to 2.745 | ns |
| 1 vs. 10 | 1.310 | -0.4297 to 3.050 | ns |
| 1 vs. 11 | 1.050 | -0.7952 to 2.895 | ns |
| 1 vs. 12 | 1.330 | -0.4574 to 3.117 | ns |
| 2 vs. 3 | -0.4700 | -2.571 to 1.631 | ns |
| 2 vs. 4 | 0.2200 | -1.725 to 2.165 | ns |
| 2 vs. 5 | -0.9900 | -2.835 to 0.8552 | ns |
| 2 vs. 6 | -0.2300 | -2.175 to 1.715 | ns |
| 2 vs. 7 | -0.08000 | -1.925 to 1.765 | ns |
| 2 vs. 8 | -0.8600 | -2.805 to 1.085 | ns |
| 2 vs. 9 | -0.6100 | -2.555 to 1.335 | ns |
| 2 vs. 10 | -0.2000 | -2.045 to 1.645 | ns |
| 2 vs. 11 | -0.4600 | -2.405 to 1.485 | ns |
| 2 vs. 12 | -0.1800 | -2.070 to 1.710 | ns |
| 3 vs. 4 | 0.6900 | -1.411 to 2.791 | ns |
| 3 vs. 5 | -0.5200 | -2.529 to 1.489 | ns |
| 3 vs. 6 | 0.2400 | -1.861 to 2.341 | ns |
| 3 vs. 7 | 0.3900 | -1.619 to 2.399 | ns |
| 3 vs. 8 | -0.3900 | -2.491 to 1.711 | ns |
| 3 vs. 9 | -0.1400 | -2.241 to 1.961 | ns |
| 3 vs. 10 | 0.2700 | -1.739 to 2.279 | ns |
| 3 vs. 11 | 0.01000 | -2.091 to 2.111 | ns |
| 3 vs. 12 | 0.2900 | -1.760 to 2.340 | ns |
| 4 vs. 5 | -1.210 | -3.055 to 0.6352 | ns |
| 4 vs. 6 | -0.4500 | -2.395 to 1.495 | ns |
| 4 vs. 7 | -0.3000 | -2.145 to 1.545 | ns |
| 4 vs. 8 | -1.080 | -3.025 to 0.8650 | ns |
| 4 vs. 9 | -0.8300 | -2.775 to 1.115 | ns |
| 4 vs. 10 | -0.4200 | -2.265 to 1.425 | ns |
| 4 vs. 11 | -0.6800 | -2.625 to 1.265 | ns |
| 4 vs. 12 | -0.4000 | -2.290 to 1.490 | ns |
| 5 vs. 6 | 0.7600 | -1.085 to 2.605 | ns |
| 5 vs. 7 | 0.9100 | -0.8297 to 2.650 | ns |
| 5 vs. 8 | 0.1300 | -1.715 to 1.975 | ns |
| 5 vs. 9 | 0.3800 | -1.465 to 2.225 | ns |
| 5 vs. 10 | 0.7900 | -0.9497 to 2.530 | ns |
| 5 vs. 11 | 0.5300 | -1.315 to 2.375 | ns |
| 5 vs. 12 | 0.8100 | -0.9774 to 2.597 | ns |
| 6 vs. 7 | 0.1500 | -1.695 to 1.995 | ns |
| 6 vs. 8 | -0.6300 | -2.575 to 1.315 | ns |
| 6 vs. 9 | -0.3800 | -2.325 to 1.565 | ns |
| 6 vs. 10 | 0.03000 | -1.815 to 1.875 | ns |
| 6 vs. 11 | -0.2300 | -2.175 to 1.715 | ns |
| 6 vs. 12 | 0.05000 | -1.840 to 1.940 | ns |
| 7 vs. 8 | -0.7800 | -2.625 to 1.065 | ns |
| 7 vs. 9 | -0.5300 | -2.375 to 1.315 | ns |
| 7 vs. 10 | -0.1200 | -1.860 to 1.620 | ns |
| 7 vs. 11 | -0.3800 | -2.225 to 1.465 | ns |
| 7 vs. 12 | -0.1000 | -1.887 to 1.687 | ns |
| 8 vs. 9 | 0.2500 | -1.695 to 2.195 | ns |
| 8 vs. 10 | 0.6600 | -1.185 to 2.505 | ns |
| 8 vs. 11 | 0.4000 | -1.545 to 2.345 | ns |
| 8 vs. 12 | 0.6800 | -1.210 to 2.570 | ns |
| 9 vs. 10 | 0.4100 | -1.435 to 2.255 | ns |
| 9 vs. 11 | 0.1500 | -1.795 to 2.095 | ns |
| 9 vs. 12 | 0.4300 | -1.460 to 2.320 | ns |
| 10 vs. 11 | -0.2600 | -2.105 to 1.585 | ns |
| 10 vs. 12 | 0.02000 | -1.767 to 1.807 | ns |
| 11 vs. 12 | 0.2800 | -1.610 to 2.170 | ns |

**Supplementary material Table S2.**

Statistical details for Figure S2 (nutrient concentrations). ns, not significant; * p<0.05; ** p<0.01; *** p<0.0001; **** p<0.00001.

1. Figure S2A - lipids: One-way ANOVA. Tukey’s post test.

| **Tukey's multiple comparisons test** | **Mean diff.** | **95% CI of diff.** | **Significance** |
| --- | --- | --- | --- |
| 1 vs. 2 | -0.6900 | -2.008 to 0.6279 | ns |
| 1 vs. 3 | -1.730 | -3.139 to -0.3211 | ** |
| 1 vs. 4 | -0.1700 | -1.579 to 1.239 | ns |
| 1 vs. 5 | -1.840 | -3.198 to -0.4824 | ** |
| 1 vs. 6 | -2.390 | -3.708 to -1.072 | **** |
| 1 vs. 7 | -0.9700 | -2.288 to 0.3479 | ns |
| 1 vs. 8 | -0.8000 | -2.209 to 0.6089 | ns |
| 1 vs. 9 | -1.020 | -2.338 to 0.2979 | ns |
| 1 vs. 10 | -2.180 | -3.498 to -0.8621 | **** |
| 1 vs. 11 | -0.8700 | -2.188 to 0.4479 | ns |
| 1 vs. 12 | -0.4400 | -1.758 to 0.8779 | ns |
| 2 vs. 3 | -1.040 | -2.358 to 0.2779 | ns |
| 2 vs. 4 | 0.5200 | -0.7979 to 1.838 | ns |
| 2 vs. 5 | -1.150 | -2.413 to 0.1130 | ns |
| 2 vs. 6 | -1.700 | -2.920 to -0.4799 | *** |
| 2 vs. 7 | -0.2800 | -1.500 to 0.9401 | ns |
| 2 vs. 8 | -0.1100 | -1.428 to 1.208 | ns |
| 2 vs. 9 | -0.3300 | -1.550 to 0.8901 | ns |
| 2 vs. 10 | -1.490 | -2.710 to -0.2699 | ** |
| 2 vs. 11 | -0.1800 | -1.400 to 1.040 | ns |
| 2 vs. 12 | 0.2500 | -0.9701 to 1.470 | ns |
| 3 vs. 4 | 1.560 | 0.1511 to 2.969 | * |
| 3 vs. 5 | -0.1100 | -1.468 to 1.248 | ns |
| 3 vs. 6 | -0.6600 | -1.978 to 0.6579 | ns |
| 3 vs. 7 | 0.7600 | -0.5579 to 2.078 | ns |
| 3 vs. 8 | 0.9300 | -0.4789 to 2.339 | ns |
| 3 vs. 9 | 0.7100 | -0.6079 to 2.028 | ns |
| 3 vs. 10 | -0.4500 | -1.768 to 0.8679 | ns |
| 3 vs. 11 | 0.8600 | -0.4579 to 2.178 | ns |
| 3 vs. 12 | 1.290 | -0.02790 to 2.608 | ns |
| 4 vs. 5 | -1.670 | -3.028 to -0.3124 | ** |
| 4 vs. 6 | -2.220 | -3.538 to -0.9021 | **** |
| 4 vs. 7 | -0.8000 | -2.118 to 0.5179 | ns |
| 4 vs. 8 | -0.6300 | -2.039 to 0.7789 | ns |
| 4 vs. 9 | -0.8500 | -2.168 to 0.4679 | ns |
| 4 vs. 10 | -2.010 | -3.328 to -0.6921 | *** |
| 4 vs. 11 | -0.7000 | -2.018 to 0.6179 | ns |
| 4 vs. 12 | -0.2700 | -1.588 to 1.048 | ns |
| 5 vs. 6 | -0.5500 | -1.813 to 0.7130 | ns |
| 5 vs. 7 | 0.8700 | -0.3930 to 2.133 | ns |
| 5 vs. 8 | 1.040 | -0.3176 to 2.398 | ns |
| 5 vs. 9 | 0.8200 | -0.4430 to 2.083 | ns |
| 5 vs. 10 | -0.3400 | -1.603 to 0.9230 | ns |
| 5 vs. 11 | 0.9700 | -0.2930 to 2.233 | ns |
| 5 vs. 12 | 1.400 | 0.1370 to 2.663 | * |
| 6 vs. 7 | 1.420 | 0.1999 to 2.640 | ** |
| 6 vs. 8 | 1.590 | 0.2721 to 2.908 | ** |
| 6 vs. 9 | 1.370 | 0.1499 to 2.590 | * |
| 6 vs. 10 | 0.2100 | -1.010 to 1.430 | ns |
| 6 vs. 11 | 1.520 | 0.2999 to 2.740 | ** |
| 6 vs. 12 | 1.950 | 0.7299 to 3.170 | **** |
| 7 vs. 8 | 0.1700 | -1.148 to 1.488 | ns |
| 7 vs. 9 | -0.05000 | -1.270 to 1.170 | ns |
| 7 vs. 10 | -1.210 | -2.430 to 0.01014 | ns |
| 7 vs. 11 | 0.1000 | -1.120 to 1.320 | ns |
| 7 vs. 12 | 0.5300 | -0.6901 to 1.750 | ns |
| 8 vs. 9 | -0.2200 | -1.538 to 1.098 | ns |
| 8 vs. 10 | -1.380 | -2.698 to -0.06210 | * |
| 8 vs. 11 | -0.07000 | -1.388 to 1.248 | ns |
| 8 vs. 12 | 0.3600 | -0.9579 to 1.678 | ns |
| 9 vs. 10 | -1.160 | -2.380 to 0.06014 | ns |
| 9 vs. 11 | 0.1500 | -1.070 to 1.370 | ns |
| 9 vs. 12 | 0.5800 | -0.6401 to 1.800 | ns |
| 10 vs. 11 | 1.310 | 0.08986 to 2.530 | * |
| 10 vs. 12 | 1.740 | 0.5199 to 2.960 | *** |
| 11 vs. 12 | 0.4300 | -0.7901 to 1.650 | ns |

2. Figure S2B - proteins: One-way ANOVA. Tukey’s post test.

| **Tukey's multiple comparisons test** | **Mean diff.** | **95% CI of diff.** | **Significance** |
| --- | --- | --- | --- |
| 1 vs. 2 | 0.8600 | -1.823 to 3.543 | ns |
| 1 vs. 3 | 0.9500 | -1.534 to 3.434 | ns |
| 1 vs. 4 | -3.670 | -6.353 to -0.9875 | *** |
| 1 vs. 5 | -1.650 | -4.006 to 0.7061 | ns |
| 1 vs. 6 | -0.4100 | -2.766 to 1.946 | ns |
| 1 vs. 7 | 0.5000 | -2.183 to 3.183 | ns |
| 1 vs. 8 | -1.670 | -4.154 to 0.8136 | ns |
| 1 vs. 9 | -0.6500 | -3.134 to 1.834 | ns |
| 1 vs. 10 | -1.630 | -3.986 to 0.7261 | ns |
| 1 vs. 11 | 1.210 | -1.146 to 3.566 | ns |
| 1 vs. 12 | -3.350 | -5.834 to -0.8664 | ** |
| 2 vs. 3 | 0.09000 | -2.593 to 2.773 | ns |
| 2 vs. 4 | -4.530 | -7.398 to -1.662 | **** |
| 2 vs. 5 | -2.510 | -5.075 to 0.05501 | ns |
| 2 vs. 6 | -1.270 | -3.835 to 1.295 | ns |
| 2 vs. 7 | -0.3600 | -3.228 to 2.508 | ns |
| 2 vs. 8 | -2.530 | -5.213 to 0.1526 | ns |
| 2 vs. 9 | -1.510 | -4.193 to 1.173 | ns |
| 2 vs. 10 | -2.490 | -5.055 to 0.07501 | ns |
| 2 vs. 11 | 0.3500 | -2.215 to 2.915 | ns |
| 2 vs. 12 | -4.210 | -6.893 to -1.527 | **** |
| 3 vs. 4 | -4.620 | -7.303 to -1.937 | **** |
| 3 vs. 5 | -2.600 | -4.956 to -0.2439 | * |
| 3 vs. 6 | -1.360 | -3.716 to 0.9961 | ns |
| 3 vs. 7 | -0.4500 | -3.133 to 2.233 | ns |
| 3 vs. 8 | -2.620 | -5.104 to -0.1364 | * |
| 3 vs. 9 | -1.600 | -4.084 to 0.8836 | ns |
| 3 vs. 10 | -2.580 | -4.936 to -0.2239 | * |
| 3 vs. 11 | 0.2600 | -2.096 to 2.616 | ns |
| 3 vs. 12 | -4.300 | -6.784 to -1.816 | **** |
| 4 vs. 5 | 2.020 | -0.5450 to 4.585 | ns |
| 4 vs. 6 | 3.260 | 0.6950 to 5.825 | ** |
| 4 vs. 7 | 4.170 | 1.302 to 7.038 | *** |
| 4 vs. 8 | 2.000 | -0.6826 to 4.683 | ns |
| 4 vs. 9 | 3.020 | 0.3375 to 5.703 | * |
| 4 vs. 10 | 2.040 | -0.5250 to 4.605 | ns |
| 4 vs. 11 | 4.880 | 2.315 to 7.445 | **** |
| 4 vs. 12 | 0.3200 | -2.363 to 3.003 | ns |
| 5 vs. 6 | 1.240 | -0.9814 to 3.461 | ns |
| 5 vs. 7 | 2.150 | -0.4150 to 4.715 | ns |
| 5 vs. 8 | -0.02000 | -2.376 to 2.336 | ns |
| 5 vs. 9 | 1.000 | -1.356 to 3.356 | ns |
| 5 vs. 10 | 0.02000 | -2.201 to 2.241 | ns |
| 5 vs. 11 | 2.860 | 0.6386 to 5.081 | ** |
| 5 vs. 12 | -1.700 | -4.056 to 0.6561 | ns |
| 6 vs. 7 | 0.9100 | -1.655 to 3.475 | ns |
| 6 vs. 8 | -1.260 | -3.616 to 1.096 | ns |
| 6 vs. 9 | -0.2400 | -2.596 to 2.116 | ns |
| 6 vs. 10 | -1.220 | -3.441 to 1.001 | ns |
| 6 vs. 11 | 1.620 | -0.6014 to 3.841 | ns |
| 6 vs. 12 | -2.940 | -5.296 to -0.5839 | ** |
| 7 vs. 8 | -2.170 | -4.853 to 0.5125 | ns |
| 7 vs. 9 | -1.150 | -3.833 to 1.533 | ns |
| 7 vs. 10 | -2.130 | -4.695 to 0.4350 | ns |
| 7 vs. 11 | 0.7100 | -1.855 to 3.275 | ns |
| 7 vs. 12 | -3.850 | -6.533 to -1.167 | *** |
| 8 vs. 9 | 1.020 | -1.464 to 3.504 | ns |
| 8 vs. 10 | 0.04000 | -2.316 to 2.396 | ns |
| 8 vs. 11 | 2.880 | 0.5239 to 5.236 | ** |
| 8 vs. 12 | -1.680 | -4.164 to 0.8036 | ns |
| 9 vs. 10 | -0.9800 | -3.336 to 1.376 | ns |
| 9 vs. 11 | 1.860 | -0.4961 to 4.216 | ns |
| 9 vs. 12 | -2.700 | -5.184 to -0.2164 | * |
| 10 vs. 11 | 2.840 | 0.6186 to 5.061 | ** |
| 10 vs. 12 | -1.720 | -4.076 to 0.6361 | ns |
| 11 vs. 12 | -4.560 | -6.916 to -2.204 | **** |

3. Figure S2C: One-way ANOVA. Tukey’s post test.

| **Tukey's multiple comparisons test** | **Mean diff.** | **95% CI of diff.** | **Significance** |
| --- | --- | --- | --- |
| 1 vs. 2 | 0.5400 | -1.525 to 2.605 | ns |
| 1 vs. 3 | -0.9100 | -3.117 to 1.297 | ns |
| 1 vs. 4 | -2.470 | -4.677 to -0.2627 | * |
| 1 vs. 5 | 1.050 | -1.077 to 3.177 | ns |
| 1 vs. 6 | 0.5500 | -1.515 to 2.615 | ns |
| 1 vs. 7 | -1.030 | -3.095 to 1.035 | ns |
| 1 vs. 8 | 0.1300 | -2.077 to 2.337 | ns |
| 1 vs. 9 | -0.8500 | -2.915 to 1.215 | ns |
| 1 vs. 10 | -0.3900 | -2.455 to 1.675 | ns |
| 1 vs. 11 | 1.120 | -0.9448 to 3.185 | ns |
| 1 vs. 12 | -2.950 | -5.015 to -0.8852 | *** |
| 2 vs. 3 | -1.450 | -3.515 to 0.6148 | ns |
| 2 vs. 4 | -3.010 | -5.075 to -0.9452 | *** |
| 2 vs. 5 | 0.5100 | -1.469 to 2.489 | ns |
| 2 vs. 6 | 0.01000 | -1.902 to 1.922 | ns |
| 2 vs. 7 | -1.570 | -3.482 to 0.3416 | ns |
| 2 vs. 8 | -0.4100 | -2.475 to 1.655 | ns |
| 2 vs. 9 | -1.390 | -3.302 to 0.5216 | ns |
| 2 vs. 10 | -0.9300 | -2.842 to 0.9816 | ns |
| 2 vs. 11 | 0.5800 | -1.332 to 2.492 | ns |
| 2 vs. 12 | -3.490 | -5.402 to -1.578 | **** |
| 3 vs. 4 | -1.560 | -3.767 to 0.6473 | ns |
| 3 vs. 5 | 1.960 | -0.1670 to 4.087 | ns |
| 3 vs. 6 | 1.460 | -0.6048 to 3.525 | ns |
| 3 vs. 7 | -0.1200 | -2.185 to 1.945 | ns |
| 3 vs. 8 | 1.040 | -1.167 to 3.247 | ns |
| 3 vs. 9 | 0.06000 | -2.005 to 2.125 | ns |
| 3 vs. 10 | 0.5200 | -1.545 to 2.585 | ns |
| 3 vs. 11 | 2.030 | -0.03476 to 4.095 | ns |
| 3 vs. 12 | -2.040 | -4.105 to 0.02476 | ns |
| 4 vs. 5 | 3.520 | 1.393 to 5.647 | **** |
| 4 vs. 6 | 3.020 | 0.9552 to 5.085 | *** |
| 4 vs. 7 | 1.440 | -0.6248 to 3.505 | ns |
| 4 vs. 8 | 2.600 | 0.3927 to 4.807 | ** |
| 4 vs. 9 | 1.620 | -0.4448 to 3.685 | ns |
| 4 vs. 10 | 2.080 | 0.01524 to 4.145 | * |
| 4 vs. 11 | 3.590 | 1.525 to 5.655 | **** |
| 4 vs. 12 | -0.4800 | -2.545 to 1.585 | ns |
| 5 vs. 6 | -0.5000 | -2.479 to 1.479 | ns |
| 5 vs. 7 | -2.080 | -4.059 to -0.1013 | * |
| 5 vs. 8 | -0.9200 | -3.047 to 1.207 | ns |
| 5 vs. 9 | -1.900 | -3.879 to 0.07869 | ns |
| 5 vs. 10 | -1.440 | -3.419 to 0.5387 | ns |
| 5 vs. 11 | 0.07000 | -1.909 to 2.049 | ns |
| 5 vs. 12 | -4.000 | -5.979 to -2.021 | **** |
| 6 vs. 7 | -1.580 | -3.492 to 0.3316 | ns |
| 6 vs. 8 | -0.4200 | -2.485 to 1.645 | ns |
| 6 vs. 9 | -1.400 | -3.312 to 0.5116 | ns |
| 6 vs. 10 | -0.9400 | -2.852 to 0.9716 | ns |
| 6 vs. 11 | 0.5700 | -1.342 to 2.482 | ns |
| 6 vs. 12 | -3.500 | -5.412 to -1.588 | **** |
| 7 vs. 8 | 1.160 | -0.9048 to 3.225 | ns |
| 7 vs. 9 | 0.1800 | -1.732 to 2.092 | ns |
| 7 vs. 10 | 0.6400 | -1.272 to 2.552 | ns |
| 7 vs. 11 | 2.150 | 0.2384 to 4.062 | * |
| 7 vs. 12 | -1.920 | -3.832 to -0.008401 | * |
| 8 vs. 9 | -0.9800 | -3.045 to 1.085 | ns |
| 8 vs. 10 | -0.5200 | -2.585 to 1.545 | ns |
| 8 vs. 11 | 0.9900 | -1.075 to 3.055 | ns |
| 8 vs. 12 | -3.080 | -5.145 to -1.015 | *** |
| 9 vs. 10 | 0.4600 | -1.452 to 2.372 | ns |
| 9 vs. 11 | 1.970 | 0.05840 to 3.882 | * |
| 9 vs. 12 | -2.100 | -4.012 to -0.1884 | * |
| 10 vs. 11 | 1.510 | -0.4016 to 3.422 | ns |
| 10 vs. 12 | -2.560 | -4.472 to -0.6484 | ** |
| 11 vs. 12 | -4.070 | -5.982 to -2.158 | **** |

4. Figure S2D: One-way ANOVA. Tukey’s post test.

| **Tukey's multiple comparisons test** | **Mean diff.** | **95% CI of diff.** | **Significant** |
| --- | --- | --- | --- |
| 1 vs. 2 | 0.01000 | -0.2527 to 0.2727 | ns |
| 1 vs. 3 | -0.1900 | -0.4738 to 0.09378 | ns |
| 1 vs. 4 | -0.1000 | -0.3627 to 0.1627 | ns |
| 1 vs. 5 | -0.2400 | -0.5027 to 0.02273 | ns |
| 1 vs. 6 | -0.1700 | -0.4327 to 0.09273 | ns |
| 1 vs. 7 | -0.1200 | -0.3827 to 0.1427 | ns |
| 1 vs. 8 | -0.1500 | -0.4127 to 0.1127 | ns |
| 1 vs. 9 | -0.1300 | -0.3927 to 0.1327 | ns |
| 1 vs. 10 | -0.1200 | -0.3827 to 0.1427 | ns |
| 1 vs. 11 | 0.0300 | -0.2538 to 0.3138 | ns |
| 1 vs. 12 | 0.0500 | -0.1993 to 0.2993 | ns |
| 2 vs. 3 | -0.2000 | -0.4838 to 0.08378 | ns |
| 2 vs. 4 | -0.1100 | -0.3727 to 0.1527 | ns |
| 2 vs. 5 | -0.2500 | -0.5127 to 0.01273 | ns |
| 2 vs. 6 | -0.1800 | -0.4427 to 0.08273 | ns |
| 2 vs. 7 | -0.1300 | -0.3927 to 0.1327 | ns |
| 2 vs. 8 | -0.1600 | -0.4227 to 0.1027 | ns |
| 2 vs. 9 | -0.1400 | -0.4027 to 0.1227 | ns |
| 2 vs. 10 | -0.1300 | -0.3927 to 0.1327 | ns |
| 2 vs. 11 | 0.0200 | -0.2638 to 0.3038 | ns |
| 2 vs. 12 | 0.04000 | -0.2093 to 0.2893 | ns |
| 3 vs. 4 | 0.09000 | -0.1938 to 0.3738 | ns |
| 3 vs. 5 | -0.05000 | -0.3338 to 0.2338 | ns |
| 3 vs. 6 | 0.02000 | -0.2638 to 0.3038 | ns |
| 3 vs. 7 | 0.07000 | -0.2138 to 0.3538 | ns |
| 3 vs. 8 | 0.04000 | -0.2438 to 0.3238 | ns |
| 3 vs. 9 | 0.06000 | -0.2238 to 0.3438 | ns |
| 3 vs. 10 | 0.07000 | -0.2138 to 0.3538 | ns |
| 3 vs. 11 | 0.2200 | -0.08338 to 0.5234 | ns |
| 3 vs. 12 | 0.2400 | -0.03135 to 0.5113 | ns |
| 4 vs. 5 | -0.1400 | -0.4027 to 0.1227 | ns |
| 4 vs. 6 | -0.07000 | -0.3327 to 0.1927 | ns |
| 4 vs. 7 | -0.02000 | -0.2827 to 0.2427 | ns |
| 4 vs. 8 | -0.05000 | -0.3127 to 0.2127 | ns |
| 4 vs. 9 | -0.0300 | -0.2927 to 0.2327 | ns |
| 4 vs. 10 | -0.02000 | -0.2827 to 0.2427 | ns |
| 4 vs. 11 | 0.1300 | -0.1538 to 0.4138 | ns |
| 4 vs. 12 | 0.1500 | -0.09925 to 0.3993 | ns |
| 5 vs. 6 | 0.07000 | -0.1927 to 0.3327 | ns |
| 5 vs. 7 | 0.1200 | -0.1427 to 0.3827 | ns |
| 5 vs. 8 | 0.0900 | -0.1727 to 0.3527 | ns |
| 5 vs. 9 | 0.1100 | -0.1527 to 0.3727 | ns |
| 5 vs. 10 | 0.1200 | -0.1427 to 0.3827 | ns |
| 5 vs. 11 | 0.2700 | -0.01378 to 0.5538 | ns |
| 5 vs. 12 | 0.2900 | 0.04075 to 0.5393 | ** |
| 6 vs. 7 | 0.05000 | -0.2127 to 0.3127 | ns |
| 6 vs. 8 | 0.02000 | -0.2427 to 0.2827 | ns |
| 6 vs. 9 | 0.04000 | -0.2227 to 0.3027 | ns |
| 6 vs. 10 | 0.05000 | -0.2127 to 0.3127 | ns |
| 6 vs. 11 | 0.2000 | -0.08378 to 0.4838 | ns |
| 6 vs. 12 | 0.2200 | -0.02925 to 0.4693 | ns |
| 7 vs. 8 | -0.0300 | -0.2927 to 0.2327 | ns |
| 7 vs. 9 | -0.01000 | -0.2727 to 0.2527 | ns |
| 7 vs. 10 | 0.0 | -0.2627 to 0.2627 | ns |
| 7 vs. 11 | 0.1500 | -0.1338 to 0.4338 | ns |
| 7 vs. 12 | 0.1700 | -0.07925 to 0.4193 | ns |
| 8 vs. 9 | 0.02000 | -0.2427 to 0.2827 | ns |
| 8 vs. 10 | 0.0300 | -0.2327 to 0.2927 | ns |
| 8 vs. 11 | 0.1800 | -0.1038 to 0.4638 | ns |
| 8 vs. 12 | 0.2000 | -0.04925 to 0.4493 | ns |
| 9 vs. 10 | 0.01000 | -0.2527 to 0.2727 | ns |
| 9 vs. 11 | 0.1600 | -0.1238 to 0.4438 | ns |
| 9 vs. 12 | 0.1800 | -0.06925 to 0.4293 | ns |
| 10 vs. 11 | 0.1500 | -0.1338 to 0.4338 | ns |
| 10 vs. 12 | 0.1700 | -0.07925 to 0.4193 | ns |
| 11 vs. 12 | 0.0200 | -0.2513 to 0.2913 | ns |
